# Waveguide Integrated Self-Powered MoS_2_ Photodetectors in the Shortwave Infrared Wavelengths

**DOI:** 10.1021/acsphotonics.5c01893

**Published:** 2025-10-20

**Authors:** Eitan Kaminski, Nathan Suleymanov, Boris Minkovich, Anastasios Polymerakis, Liana Kartvelishvili, Vladislav Kostianovski, Eilam Yalon, Elefterios Lidorikis, Ilya Goykhman

**Affiliations:** † Micro Nanoelectronics Research Center, Department of Electrical and Computer Engineering, Technion, Haifa 32000, Israel; ‡ Institute of Applied Physics, The Faculty of Science and The Center for Nanoscience and Nanotechnology, 26742 The Hebrew University of Jerusalem, Jerusalem 91904, Israel; § Department of Materials Science and Engineering, University of Ioannina, 45110 Ioannina, Greece

**Keywords:** 2D materials, waveguide integrated photodetectors, zero-bias photodetection, photothermoelectric effect

## Abstract

Broadband photodetectors (PDs) are essential for various applications, including optical communication, sensing, and imaging. Modern semiconductor PD technologies often face challenges related to spectral coverage, power consumption, complex manufacturing, and limited integration with silicon electronics. As photonics technologies continue to advance alongside growing performance demands, exploring new avenues for innovative, cost-effective broadband PDs with reduced power consumption and manufacturing complexity is becoming increasingly important. In this work, we present a zero-bias, waveguide-integrated PD based on single-layer MoS_2_, which operates at telecom wavelengths with no dark current. By utilizing the photothermoelectric effect combined with internal photoemission process, our devices demonstrate a record responsivity of ∼180 V/W at 1550 nm, the highest reported in the literature for unbiased 2D PDs operating in the short-wave infrared. The recorded frequency response is in the millisecond range, limited by the electrical RC time constant. The PD noise equivalent power is ∼500 nW at 1 Hz, dominated by 1/*f* noise, and is reduced to ∼0.3 nW at the Johnson limit. Consequently, the specific detectivity (D*) is estimated to be ∼10^5^ Jones at the 1/*f* limit, reaching ∼2 × 10^10^ Jones at Johnson noise-limited operation. Our findings contribute to developing high-efficiency broadband MoS_2_ PDs and emphasize the potential of 2D semiconductors in advancing self-powered PDs technology.

## Graphical abstract

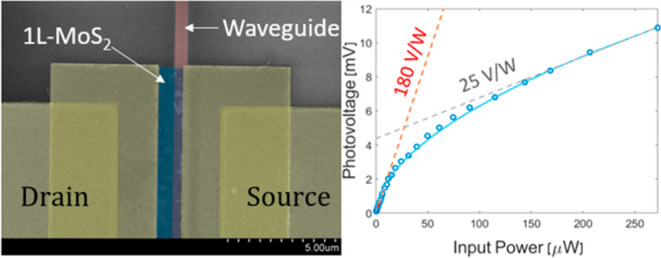

## Introduction

Broadband photodetectors (PDs) that operate across a wide range of wavelengths are essential components in modern photonic integrated circuits (PICs). These devices play a vital role in various application domains, including data communication,^1,2^ sensing,^3,4^ spectroscopy,^5,6^ and imaging,^7–10^ where high-performance room-temperature (RT) photodetection across visible and infrared spectra is required.^7,11,12^ However, current semiconductor PD technologies encounter challenges related to spectral limitations,^13^ increased power consumption,^14^ noise figures,^15^ and the incompatibility of compound semiconducting materials (e.g., InGaAs, InSb, HgCdTe) with established complementary metal-oxide-semiconductor (CMOS) fabrication facilities.^13,16^ The latter hinders scalability, increases costs, and complicates integration with modern silicon PIC and CMOS read-out electronics. As photonics technologies advance with growing performance demands, overcoming these limitations is crucial for enabling more efficient, cost-effective, and scalable PD-integrated systems. Therefore, innovative PD technologies that provide a broadband response while reducing power consumption and manufacturing complexity are increasingly necessary.

To address these limitations, over the past decade, two-dimensional (2D) materials have been increasingly explored for PD applications,^17^ revealing new opportunities for the development of novel quantum (photon)^18^ and thermal (power)^19^ detectors that outperform conventional semiconductor devices. Due to their unique optoelectronic properties, 2D semimetallic single-layer graphene (SLG) with a zero energy bandgap^20^ and 2D semiconducting transition metal dichalcogenides (TMDs) with varying bandgaps^21^ have emerged as a promising platform for realizing advanced PDs that exceed state-of-the-art performance.^22^ Furthermore, the ability to transfer 2D materials onto various substrates^23^ has opened up unique opportunities for the heterogeneous integration of 2D devices with CMOS electronics^24^ and silicon photonics (SiPh) integrated circuits. In this regard, the development of scalable, energy efficient PDs capable of operating without an external voltage bias, with reduced power consumption while maintaining high responsivity across a broad spectral range is highly desired.^25^

Among 2D materials, graphene PDs (GPDs) demonstrate an unparalleled multispectral response spanning from visible to terahertz wavelengths.^20^ Various photodetection mechanisms have been explored for effective unbiased operation, including photothermoelectric (PTE),^26^ internal photoemission (IPE),^27^ and photothermionic (PTh) effects.^28^ Notably, PTE-based GPDs ideally operate in voltage mode, enabling a direct photovoltage generation (*V*_ph_) which eliminates the need for transimpedance amplifiers (TIA) in the read-out electronics, thereby minimizing power consumption and the overall system footprint.^29^ When optically illuminated, the fast (∼fs) thermalization of photoexcited carriers in SLG through electron–electron scattering,^30^ combined with significantly slower (∼ps) heat dissipation to the lattice via phonon or defect-mediated cooling,^31^ leads to a “hot” carrier distribution in graphene where electronic temperatures rise considerably above that of the lattice. In this hyperthermal regime, *V*_ph_ is generated via the Seebeck effect if an electronic temperature gradient exists between the PD leads.^32^ This distinct hot-carrier dynamics in SLG yields voltage responsivity *R*_V_ = *V*_ph_/*P*_in_, where *P*_in_ is the incident optical power. The PTE effect has been successfully demonstrated in SLG p–n junction PDs under free-space illumination at mid-infrared wavelengths (MIR, 6–10 μm)^33^ and terahertz frequencies (1–4 THz),^34^ as well as in guided mode configurations at telecom wavelengths (1.5–1.6 μm) by integrating PTE-GPDs with photonic crystals,^35^ slot waveguides,^36^ hybrid plasmonic waveguides,^37^ and coupling to microring resonators. The latter results in a state-of-the-art *R*_V_ ∼ 90 V/W.^29^

Along with the PTE effect, contacting graphene with semiconductors (SC) creates a Schottky contact with rectifying electrical characteristics (a diode) that can function as an unbiased PD due to the built-in electric field formed across the SLG/SC interface.^38^ When operating in the IPE mode, Schottky diodes can detect sub-bandgap radiation, where photons with energy *E*_ph_ below the SC bandgap *E*_g_ (i.e.,*E*_ph_ < *E*_g_) are absorbed in SLG (i.e., conductor) and excite charge carriers above the Schottky barrier height ϕ_B_.^39^ These “hot” carriers can then be emitted into the SC with quantum yield $Y\propto {\left(E_{ph}-\phi_{B}\right)}^{2}/{\left(E_{ph}\right)}^{2}$ (Fowler equation), thus generating a photocurrent resulting in current responsivity *R*_I_ = *I*_ph_/*P*_in_. SLG/Si Schottky detectors have been demonstrated in free-space^40^ and waveguide^41^ configurations, showing unbiased *R*_I_ ∼ 20 mA/W at telecom wavelengths.^27^ Additionally, the photoresponse can be extended to mid-infrared spectra by exploiting the PTh regime,^42^ where low-energy photons (i.e.,*E*_ph_<ϕ_B_ < *E*_g_) absorbed in SLG yield hot carriers. These do not have sufficient energy to overcome ϕ_B_ directly, instead, they multiply^43^ and thermalize, gaining enough thermal energy for excess thermionic emission over ϕ_B_, resulting in *R*_I_ ∼ 100 mA/W for zero-bias operation at 3 μm wavelength.^44^

Although SLG is an attractive platform for PTE PDs, its responsivity is fundamentally limited by a moderate Seebeck coefficient.^45^ The Seebeck coefficient *S* quantifies the voltage generated in response to a temperature gradient Δ*T* (i.e., *V*_ph_ = *S*·Δ*T*), and it is closely linked to the difference between the Fermi level *E*_F_ and the average energy ⟨*E*⟩ at which charge carriers contribute to electrical conduction.^46^ According to the Mott relation,^47^ *S* is proportional to the energy scaling factor (⟨*E*⟩ – *E*_F_)/*qT*, where *q* is the electron charge and *T* is the temperature. In metals (e.g., graphene), *E*_F_ resides within the band, and only charge carriers within a narrow (∼*k*_B_ *T*) energy window around *E*_F_ participate in transport, where *k*_B_ is the Boltzmann constant. As a result, in SLG, a minor separation between ⟨*E*⟩ of conducting electrons (holes) close to *E*_F_, together with weak energy dependence of conductivity due to linear band structure, leads to moderate *S* ∼ 100 μV/K.^48^

On the other hand, semiconducting 2D TMDs, such as single-layer MoS_2_ (1L-MoS_2_), can offer an advantage in terms of thermoelectric properties. In these materials, *E*_F_ lies within the bandgap, substantially separating the Fermi level from the conduction and valence band edges, leading to a larger ⟨*E*⟩ – *E*_F_ ≫ *k*_B_ *T*. Furthermore, the sharp onset of the density of states and strong energy dependence of conductivity near the band edge increases *S*, often reaching 100–1000 μV/K^49,50^ and even higher reported values in the mW/K regime.^51,52^ This fundamental property implies better thermoelectric performance of 2D semiconductors than SLG or metals, making them an attractive candidate for developing high-efficiency PTE devices. Furthermore, a Schottky contact between 2D TMDs and metals^53^ can facilitate an additional contribution from the IPE process to enhance the overall photoresponse.

In this work, we present the design, fabrication, and characterization of waveguide-integrated unbiased 1L-MoS_2_ PDs, operating at telecom wavelengths with no dark current. We employed a 1L-MoS_2_ for its higher Seebeck coefficient compared to SLG^52^ and the ability to form a Schottky contact with metal. The device is implemented with an asymmetric contact arrangement (Figure 1) to benefit from both PTE and IPE processes. It features a simple Au/1L-MoS_2_/Au configuration, coupled to a standard silicon-on-insulator (SOI) waveguide. The realized PDs demonstrate maximum responsivity *R*_V_ ∼ 180 V/W, the highest reported in the literature for zero-bias 2D PDs at short-wave infrared (SWIR) range. To our knowledge, this is the first demonstration of a self-powered waveguide-integrated PD utilizing 2D TMD material. The obtained high *R*_V_ can be described by the combined effect of the PTE process due to the temperature gradient in the MoS_2_ channel, along with the IPE process of hot electrons’ injection across the gold Au/MoS_2_ interface. The PD response time is in the millisecond regime with a 3 dB roll-off frequency of ∼250 Hz (setup limited). The measured noise equivalent power, NEP ∼500 nW at 1 Hz, is governed by 1/f noise. The Johnson noise limited NEP is ∼0.5 nW. The response time and noise figures can be improved by increasing the conductivity (doping) of the 1L-MoS_2_ channel and optimizing the electrical and thermal contact resistance. Our results contribute to developing advanced, broadband, on-chip integrated MoS_2_ PDs with spectral response from visible to infrared wavelengths.

**Figure 1:**
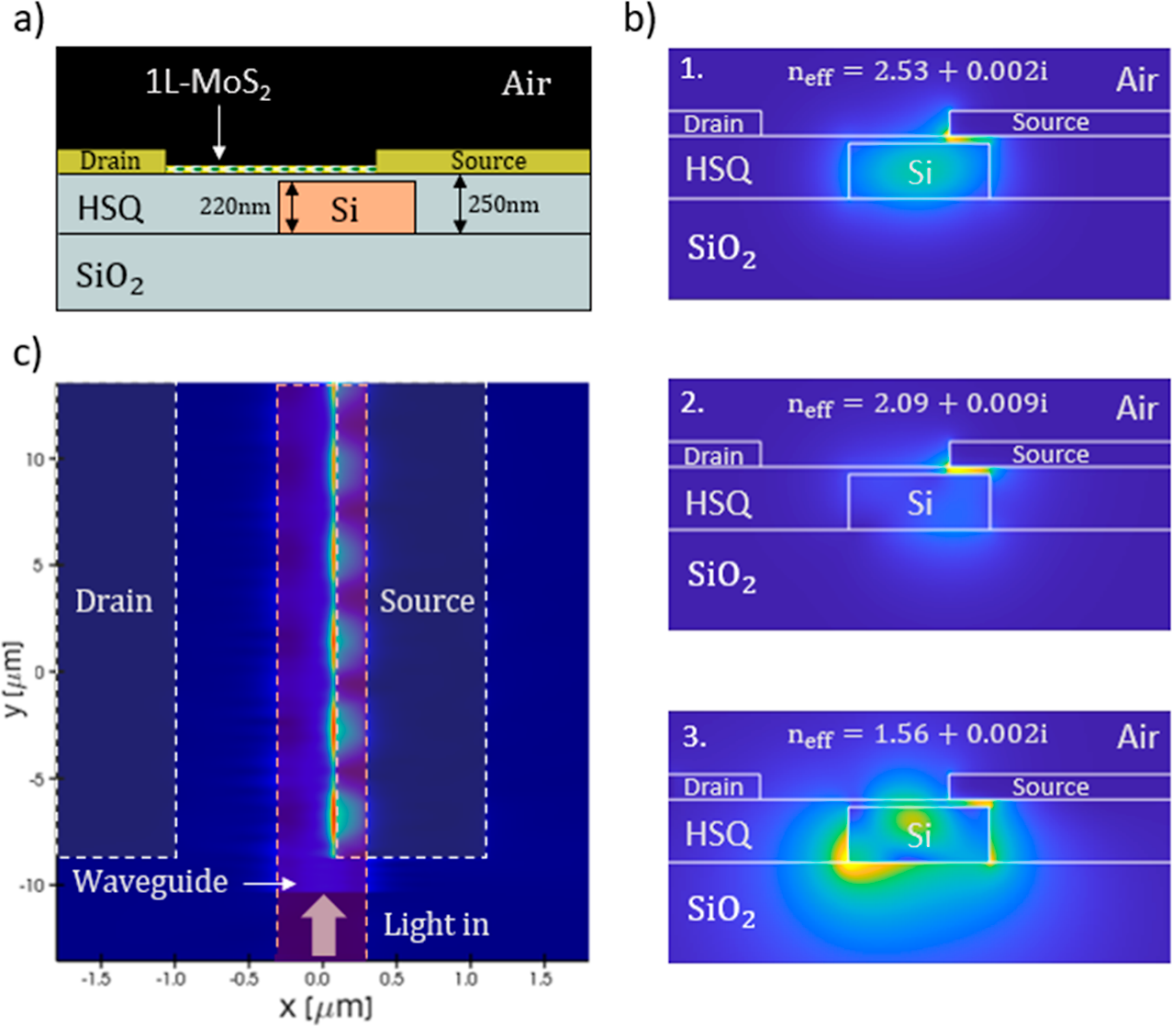
(a) Schematic device configuration featuring Au/1L-MoS_2_/Au PD asymmetrically coupled to Si waveguide to enhance PTE and IPE effects. (b) Optical intensity mode profiles supported by the device structure calculated by finite difference electromagnetic (MODE) simulations, namely (1) transverse electric (TE), (2) transverse magnetic (TM), and (3) hybrid TE–TM mode, with effective refractive indices *n*_eff_ noted @1550 nm. (c) Full 3D finite-difference time-domain (FDTD) simulation of the optical intensity of guided modes in the structure (top view) shows the beating between the Si waveguide and hybrid plasmonic modes.

## Results and Discussion

### Design, Simulation and Fabrication

Figure 1a shows the schematic structure of our PD, which consists of a chemically vapor deposition (CVD) grown 1L-MoS_2_ channel clamped between two Au contact pads, asymmetrically aligned relative to the Si waveguide.

The realized asymmetric configuration provides dual functionality. First, the plasmonic coupling induces localized heating at the source contact, which by heat conduction raise the lattice temperature of MoS_2_ in touch. Combined with the distant “cold” drain contact, the resulting temperature gradient generates *V*_ph_ across the MoS_2_ channel via the PTE effect. In addition, this SPP driven light absorption at the Au/MoS_2_ interface excites electrons above the Schottky barrier contributing to photoresponse via the IPE process. Combining these two effects in a single device enhances the device’s responsivity at the telecom wavelengths, even though these wavelengths are below the bandgap and cannot be absorbed directly in 1L-MoS_2_.^54^

First, to study the optical properties of our waveguide-integrated PD, we conducted finite-element MODE analysis and full 3D FDTD electromagnetic simulations (see Methods). Figure 1b summarizes the simulation results, showing three guided modes supported by the PD structure. Namely, the transverse electric (TE) mode, indicative of dielectric guiding (Figure 1b_1_), the transverse magnetic (TM) mode, representative of plasmonic guiding (Figure 1b_2_), and a hybrid TE and TM mode (Figure 1b_3_). The plasmonic mode is confined within the low-index SiO_2_ gap separating the Au contact and Si waveguide (Figure 1b_2_), exhibiting increased optical intensity and enhanced metal-induced absorption with a higher imaginary part of the effective refractive index. Figure 1c shows the FDTD simulation results of optical guiding in the PD (top view) upon TE-polarized incident light, revealing the beating (interference pattern) between the modes. Furthermore, using FDTD simulations, we quantified the coupling efficiency of the Si waveguide mode to the PD, evaluating the modal content and the contribution of each mode to the device’s optical absorption and responsivity (see Supporting Information, Section S1).

For the fabrication, we utilized a standard SOI substrate consisting of a 220 nm silicon device layer on top of a 2 μm buried oxide (BOX). The waveguide’s geometry (220 nm height, 560 nm width) was defined by e-beam lithography, followed by fluorine chemistry-based reactive ion etching (RIE). To realize a smooth surface on the photonic chip before the 1L-MoS2 transfer, we spin-coated and thermally annealed hydrogen silsesquioxane (HSQ) e-beam resist (see Methods). This procedure resulted in a planarized surface featuring a shallow 30 nm thick HSQ layer atop the silicon waveguide. The 1L-MoS_2_ was CVD-grown on sapphire using solid precursors. Subsequently, it was transferred onto the planarized silicon waveguide using a vacuum-assisted semidry transfer process (see Methods). To form the Au/1L-MoS_2_/Au PD, we performed additional e-beam lithography and RIE steps to shape and etch the MoS_2_ channel, followed by electron-gun-assisted deposition of Au pads to create the electrical contacts. The realized PD width and length are *W* = 1.5 μm and *L* = 25 μm, respectively. The photonic layout relies on a Y-splitter (3 dB) configuration, where the active arm accommodates the PD under test for photovoltage measurements, while the reference arm monitors the optical power reaching the device (Figure 2a). We employed grating couplers to couple TE-polarized light from a tunable laser source in and out of the photonic chip. Figure 2b shows a scanning electron microscopy (SEM) micrograph of the fabricated device, highlighting the asymmetric contact configuration relative to the waveguide. Specifically, one contact (i.e., the source) is positioned to overlap directly with the Si waveguide mode, ensuring plasmonic coupling and enhanced light absorption in the metal. In contrast, the other contact (i.e., the drain) is strategically placed away from the waveguide, minimizing its interaction with the optical mode.

**Figure 2:**
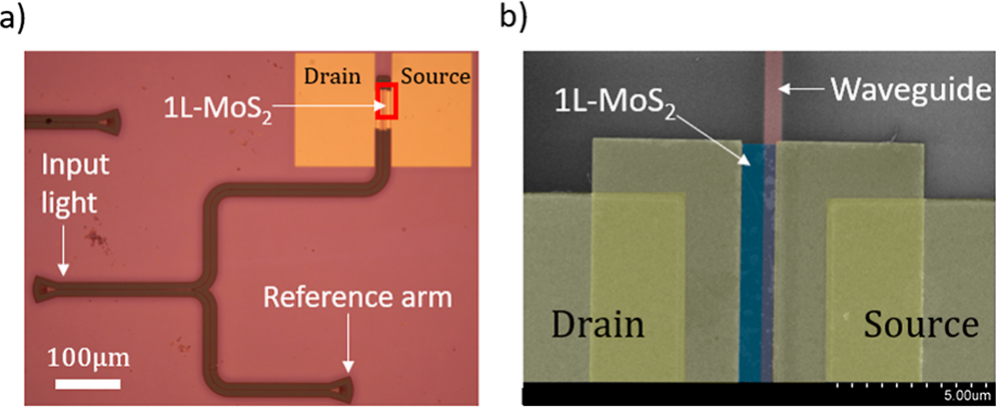
(a) An optical microscope image of the fabricated device including the Y-splitter, the integrated PD in the active (upper) arm, and the reference (lower) arm for optical power monitoring. (b) SEM micrograph (false colors) focusing on the area within the red rectangle in (a), highlighting Si waveguide, 1L-MoS_2_ layer, and asymmetric source and drain contacts relative to the waveguide.

### Material Characterizations

The quality and uniformity of the MoS_2_ layer were assessed by Raman and photoluminescence (PL) spectroscopy characterizations of the as-grown material on sapphire and after the device fabrication, where the 1L-MoS_2_ is integrated on top of the Si waveguide (see Methods). As shown in Figure 3a (red curve), the Raman spectrum of the CVD-grown MoS_2_ on sapphire under 532 nm excitation reveals two major peaks: at ∼383 cm^–1^, which corresponds to the in-plane (*E*_2g_^1^) vibrational mode, and the peak at ∼403 cm^–1^, which is associated with the out-of-plane (A_1g_) mode. The estimated full width at half-maximum (fwhm) values are ∼3.4 cm^–1^ for *E*_2g_^1^ and ∼4.8 cm^–1^ for A_1g_ respectively.^55,56^ The frequency of *E*_2g_^1^ mode decreases, whereas that of the A_1g_ mode increases with increasing layer thickness, allowing for the peak separation to serve as an indicator of layer counts.^57^ The observed peak position difference of 19.6 cm^–1^ suggests that a 1L-MoS_2_ is used in our experiments.^57^ The peak at 415 cm^–1^ (denoted by an asterisk in Figure 3a) corresponds to the A_1g_ mode of sapphire.^58^ Following the complete fabrication process, a slight red shift of <1.5 cm^–1^ is observed in the 1L-MoS_2_ Raman spectrum on SiO_2_ (i.e., HSQ optical cladding), as shown in Figure 3a (blue curve). This shift is attributed to the differences in dielectric permittivity between sapphire and SiO_2_, along with the anticipated increase in n-type doping of 1L-MoS_2_ on SiO_2_.^59,60^

**Figure 3:**
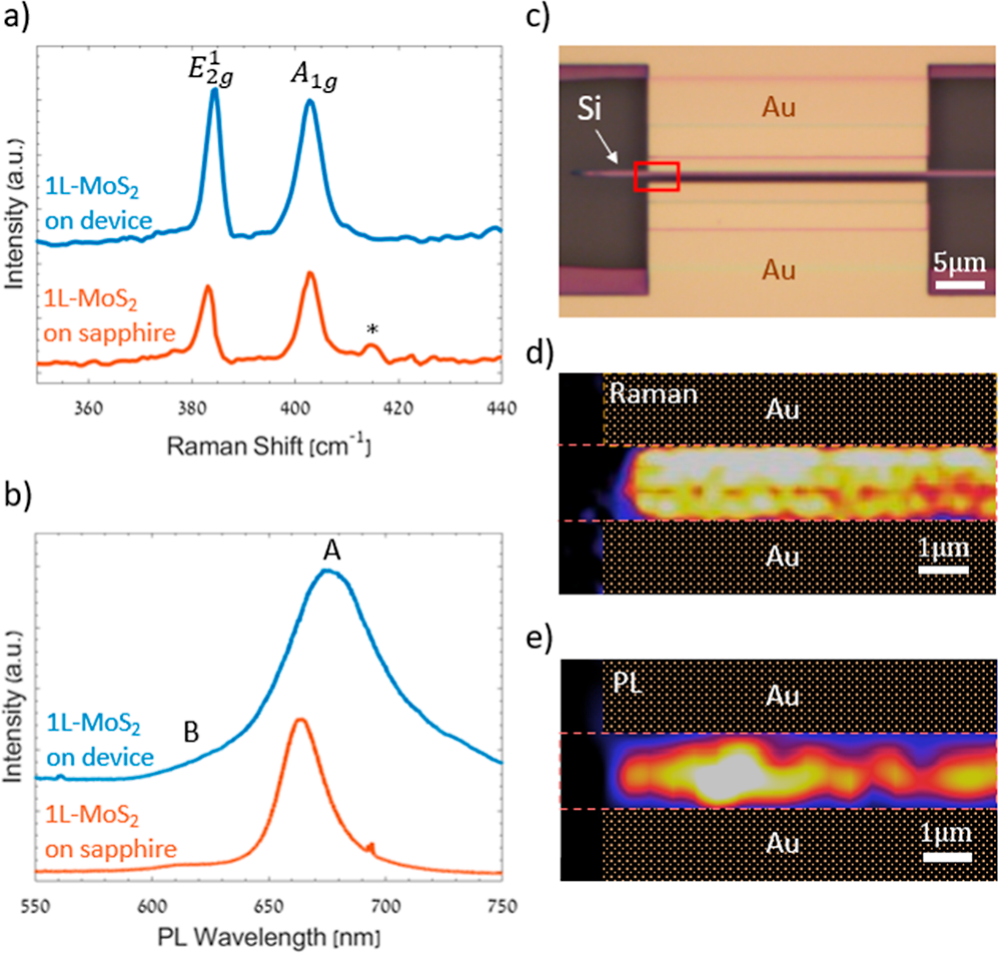
(a) Raman and (b) PL spectra of 1L-MoS_2_ at 532 nm excitation on sapphire (red line) and after device fabrication (blue line). (c) Optical microscope image of the mapped area (red rectangle) of the 1L-MoS_2_ channel of the PD. (d) Raman mapping of the A_1g_ peak showing the uniformity of the 1L-MoS_2_ PD channel. (e) PL mapping of 1L-MoS_2_ A-exciton peak indicating the spatial distribution of PL intensity and quenching in the vicinity of Au contacts.

The PL characterizations provide further evidence of 1L-MoS2 (Figure 3b). The recorded PL spectra show the characteristic excitonic content of 1L-MoS_2_,^46^ including the A- and B-exciton peaks, indicating band-to-band radiative recombination processes,^46^ i.e. A-exciton: ∼664 nm, ∼1.85 eV and B-exciton: ∼615 nm, ∼2.00 eV (see Supporting Information, Section S2). Several distinctions become apparent when comparing the PL spectra of the fabricated device (Figure 3b, blue curve) with the as-grown material on sapphire (Figure 3b, red curve). The A-exciton peak on SiO_2_ is broader and has lower intensity than on sapphire (see Supporting Information, Section S2). This is attributed to increased exciton scattering and higher n-type doping of the 1L-MoS_2_ after transfer onto SiO_2_.^61^ The latter is supported by the increased contribution of the charged exciton A^–^ (trion) emission on the PL content of the 1L-MoS_2_ on SiO_2_.^61^ The observed red shift of the exciton peaks, ∼10 nm after transfer, can be ascribed to a different dielectric environment^62^ and higher permittivity of sapphire compared to SiO_2_ substrates.^63^

To confirm the uniformity of the MoS_2_ layer after the device fabrication, we performed Raman and PL spectroscopy mapping of the PD. Figure 3d,e show representative intensity maps of 1L-MoS_2_ A_1g_ Raman peak and A-exciton PL peak collected across the PD area highlighted by a red rectangle in the optical microscope image (Figure 3c). The Raman map demonstrates a nearly uniform intensity distribution of the Raman signal in the MoS_2_ channel confined between Au contacts, confirming the uniformity of the MoS_2_ layer. Additional evidence comes from the PL signal (Figure 3e), which is enhanced in the central area above the waveguide and quenched in the vicinity of the Au contacts.

### Device Characterizations

To assess the PD responsivity, we coupled TE-polarized light from a tunable laser into the Si waveguide using a grating coupler and measured the generated *V*_ph_ between the source-drain electrodes at different *P*_in_, which were monitored by a commercial power meter in the reference arm of the Y-splitter (Figure 4a). Two distinct operational regimes are evident. In the low-power range (<20 μW in the waveguide), the peak responsivity reaches *R*_V_ ∼ 180 V/W, the highest reported in the literature for unbiased 2D PDs. For increased optical powers (>100 μW), on the other hand, the responsivity declines to *R*_V_ ∼ 25 V/W. This behavior can be attributed to several factors. Namely, a weak van der Waals (vdW) coupling and poor thermal boundary conductance (TBC ∼ 15 MW·m^–2^·K^–1^)^64^ at the Au/MoS_2_ interface can lead to a flatter temperature gradient in the MoS_2_ channel resulting in a weaker PTE effect. Moreover, concerning the IPE process, an inefficient sweep-out of photoexcited carriers from the unbiased MoS_2_ channel and corresponding space-charge-limited injection across the Au/MoS_2_ Schottky interface can restrict further photoemission from the contact at higher optical powers, reducing the total *R*_V_.

**Figure 4:**
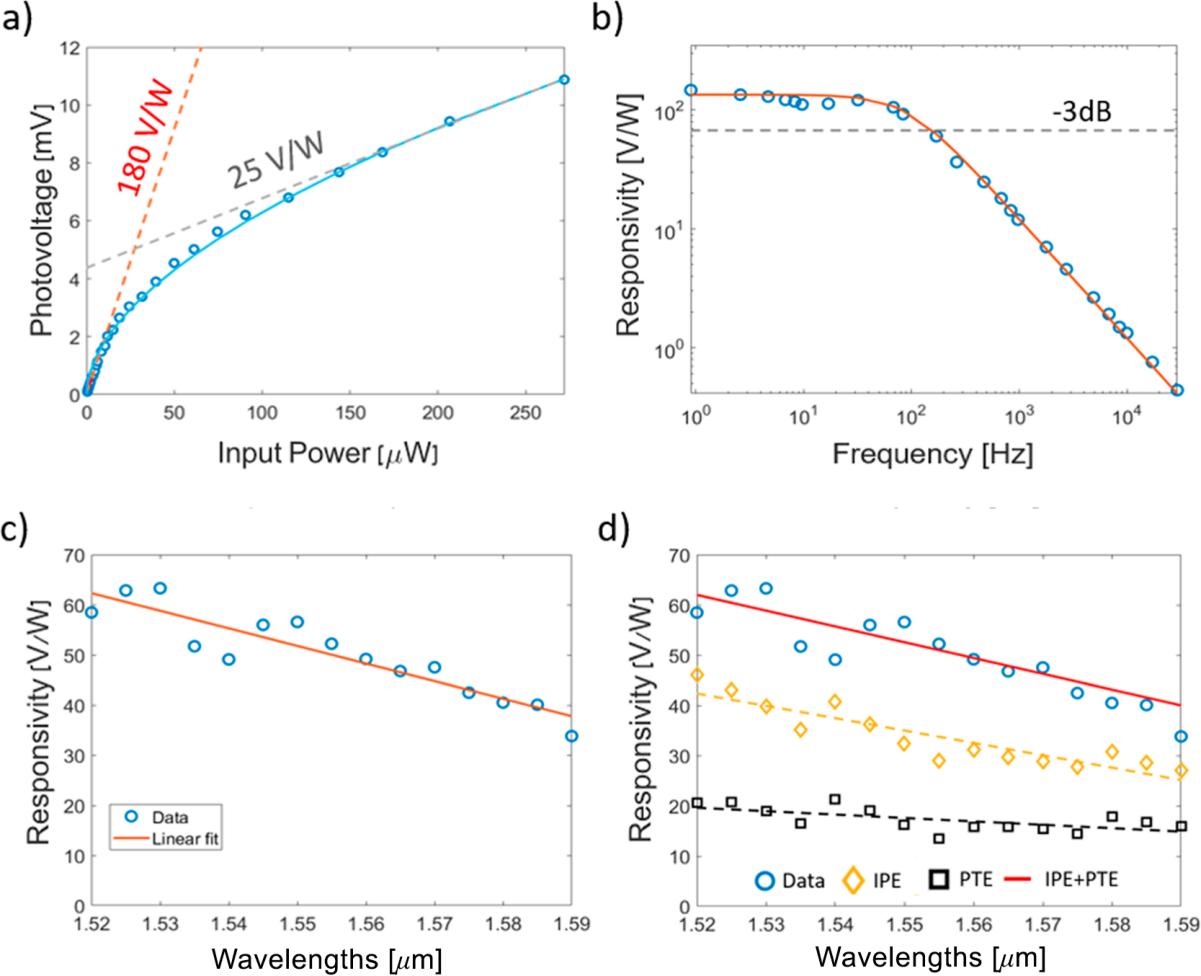
(a) Power plot of measured photovoltage at different optical powers delivered to the PD. The red and black dashed lines represent a linear fit in the low- and high-power regimes, respectively, where the slope of the fit corresponds to the PD responsivity. (b) Frequency response of the PD, with a response time in the millisecond regime and *f*_3dB_ ∼ 250 Hz. (c) Spectral response at the SWIR (1.52–1.59 μm), showing a decrease in responsivity for longer wavelengths at input power of ∼50 μW. (d) Simulated spectral response of the device using PTE and IPE processes. The combined effect (red line, IPE + PTE) demonstrates excellent agreement with the experimental data.

Figure 4b shows the frequency response of the MoS_2_ PD, with a 3 dB roll-off frequency *f*_3dB_ ∼ 250 Hz. While this appears much slower compared to graphene-based PTE devices operating at gigahertz (GHz) regime with an electronic temperature gradient,^64^ the main limiting factor in our PD is less related to the intrinsic thermal response of the MoS_2_ channel due to the lattice heating and more to the device RC time constant, i.e. *f*_3dB_ = 1/2πRC. Specifically, the 2-terminal *I*–*V* characteristics of the MoS_2_ PD exhibit rectifying electrical characteristics, indicating the formation of a Schottky junction at the Au/1L-MoS2 contacts, having a shunt resistance of *R*_Shunt_ ∼ 500 MΩ at zero voltage bias (see Supporting Information, Section S3). When measured with electrical probes having a typical parasitic capacitance of ∼1 pF, the resulting RC time constant imposes a bandwidth limit of ∼250 Hz. This implies that the measurement setup (i.e., probe capacitance) can restrain the measured *f*_3dB_. Instead, if the device’s frequency response is limited by the depletion capacitance of Au/1L-MoS2 Schottky contacts (estimated values of a few fF), the PD bandwidth is expected to be ∼300 kHz. Furthermore, the response time can be further improved by realizing an ohmic contact in the cold region, allowing the *f*_3dB_ to be governed by the MoS_2_ channel resistance *R*_ch_ ∼ 140 kΩ (see Supporting Information, Section S3), enabling the operation up to ∼100 MHz.

The spectral response of the device at SWIR is shown in Figure 4c, indicating a decrease in *R*_V_ for longer wavelengths. To validate the observed trend, we first performed comprehensive electromagnetic simulations to study the modal content of guided light reaching the PD (see Supporting Information, Section S1). Specifically, our device employs a Y-splitter configuration and utilizes grating couplers to interface with the external laser and reference power meter. Although the grating coupler is designed to launch only a TE waveguide mode, the actual optical field distribution reaching the PD may vary since the mode undergoes several bends along the waveguide path, altering the mode composition. To quantify this effect, we conducted 3D FDTD simulations to weigh the modal composition in the bus waveguide and calculate wavelength-dependent optical coupling for each mode to the PD, and evaluate their contributions to the total optical absorption (see Supporting Information, Section S1). We then used the calculated absorption values to feedback to the thermo-optic (PTE) and optoelectronic (Fowler plot, IPE) simulations and assessed the overall spectral response in our PD (see Supporting Information, Sections S4, S5). Figure 4d shows the simulated *R*_V_ with the approximate contributions of the PTE and IPE processes to the total device photoresponse. The simulated results demonstrate excellent agreement with experimental data and reproduce both the spectral trend and the *R*_V_ using realistic parameter space reported in the literature for PTE and IPE-based photodetection in 1L-MoS_2_ (see Supporting Information, Section S5).

Finally, we characterized the PD noise equivalent power ${NEP=v_{n}/R}_{V}$ (i.e., the power that gives a signal-to-noise ratio of one) where *v*_n_ is the voltage noise spectral density. The noise signal *v*_n_ was measured in the time domain, by collecting the trace on an oscilloscope, with a subsequent Fourier transform to analyze the data in the spectral domain (see Methods). Figure 5 plots the obtained noise power spectral density (PSD), indicating the 1/*f* flicker noise contribution at low (<1 kHz) frequencies and the Johnson (thermal) noise floor at higher frequencies. We then calculated *v*_n_ = (PSD)^1/2^ and used the corresponding *R*_V_ values (Figure 4b) to estimate the device NEP. As a result, we get NEP ∼ 500 nW at 1 Hz dominated by 1/*f* noise and ∼50 nW at an elevated frequency of 100 Hz. Considering the Johnson noise limit, the estimated NEP is ∼0.3 nW.

**Figure 5:**
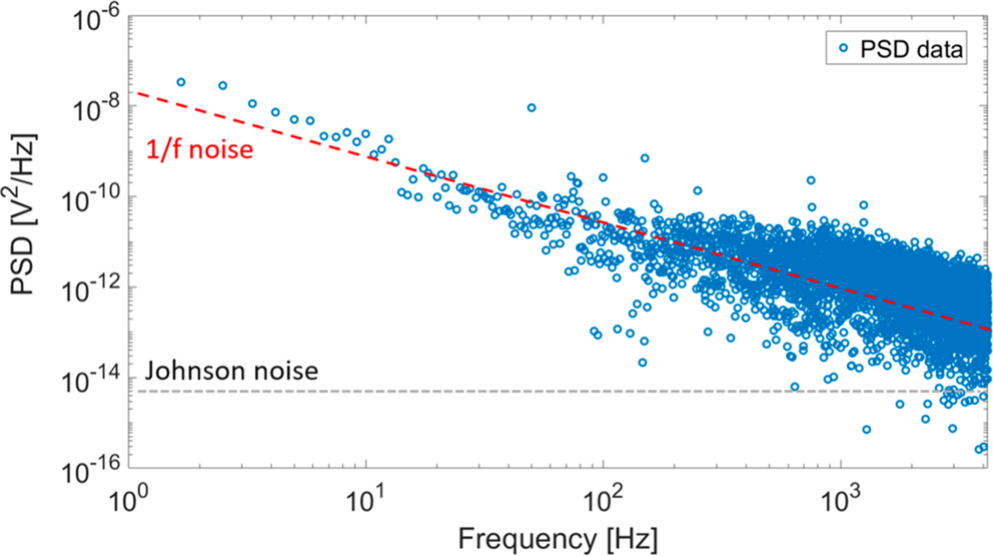
Noise power spectral density of the photodetector indicates a significant contribution of 1/*f* flicker noise at low frequencies above the Johnson noise floor.

Based on the measured NEP values, we estimated the D* of our device for 1/*f* and Johnson noise-limited operation. Specifically, for the former, we get D* ∼ 10^5^ Jones, while the latter is significantly improved, reaching D* ∼ 2 × 10^10^ Jones. This projected performance is on par with the state-of-the-art 2D PD,^65^ highlighting the potential of TMD-based self-powered infrared detectors.

## Conclusions

In summary, we demonstrated a broadband, zero-bias, waveguide-integrated PD based on 1L-MoS_2_, operating within the telecom wavelengths (1500 nm–1600 nm). The device demonstrates maximum responsivity *R*_V_ ∼ 180 V/W at optical power range <100 μW, the highest reported in the literature for zero-bias 2D PDs at short-wave infrared. This is the first demonstration of a self-powered waveguide-integrated PD utilizing 2D TMD material. The measured device roll-off frequency is *f*_3dB_ ∼ 250 Hz, primarily limited by the high ∼500 MΩ shunt resistance of the Schottky contact operating at zero bias and the setup’s limited electrical probes’ capacitance on the order of picofarad (pF). The PD frequency response can be improved to ∼100 MHz by realizing an Au/1L-MoS_2_ ohmic contact at the drain and operating in the depletion capacitance limit of the Schottky contact at the source without a substantial contribution from the probes. The measured NEP is ∼500 nW at 1 Hz, dominated by 1/*f* flicker noise, and it is reduced to 0.3 nW at the Johnson noise limit. As a result, the estimated D* for 1/*f* and Johnson noise-limited operation is ∼10^5^ Jones and ∼2 × 10^10^ Jones, respectively. Our work demonstrates the advantages of using 2D semiconductors for broadband, zero-bias photodetection, particularly in terms of responsivity and compatibility with the silicon platform. Our findings pave the way for developing highly efficient, broadband, and low-power PDs for CMOS and photonic integration.

## Methods

### Numerical Simulations

Finite-element electromagnetic simulations (MODE and 3D FDTD) were performed to characterize the optical behavior of the PDs. The simulation geometry included a silicon-on-insulator (SOI) waveguide with a 220 nm thick silicon layer (width: 560 nm, refractive index *n*_*Si*_ = 3.477 at 1550 nm)^66^ on top of a 2 μm thick buried oxide (BOX) layer (SiO_2_, *n*_SiO_^_2_^ = 1.444 at 1550 nm).^66^ A top cladding of 250 nm thick SiO_2_ was included, with air acting as the background medium. The metal contacts were modeled as gold (*n*_Au_ = 0.559 + i9.87 at 1550 nm).^66^ The 1L-MoS_2_ was not included in the purely photonic simulations, as it exhibits no optical absorption within the C-band region (1.5–1.6 μm).^54^

Photothermal simulations were performed using COMSOL Multiphysics. In these simulations, the 1L-MoS_2_ was modeled as a 0.65 nm thick layer with a thermal conductivity of 34 W/m·K.^67^ The thermal boundary conductance (TBC) at the 1L-MoS_2_/SiO_2_ interface was implemented as a boundary condition, corresponding to a reported TBC value of 15 MW·m^–2^·K^–1^.^64^ Additional details on the thermal simulations are provided in the Supporting Information, Section S4.

### Fabrication

The PDs were fabricated on a commercial SOI substrate comprising a 220 nm thick crystalline silicon device layer (p-type, resistivity 10–20 Ω·cm) on top of a 2 μm thick BOX layer. The photonic structures were patterned via electron-beam lithography (EBL) using a RAITH EBPG5200 writer and ZEP-520A e-beam resist, and subsequently etched into the silicon layer through inductively coupled plasma reactive ion etching (ICP-RIE, Plasma-Therm) using a gas mixture of C_4_F_8_/Ar/SF_6_. To achieve a planarized surface for MoS_2_ transfer, a 250 nm thick hydrogen silsesquioxane (HSQ, 0.06 XR-1541, Dow Corning) layer was spin-coated and thermally annealed at 200 °C for 30 min.

The single-layer MoS_2_ film was grown via chemical vapor deposition (CVD) on a sapphire substrate, following the method detailed in ref,^68^ producing a continuous monolayer film exceeding an area of 50 mm^2^. Transfer of the MoS_2_ film onto the photonic chip followed a modified protocol from Ref 69. Specifically, the MoS_2_ layer was first coated with a polystyrene (PS) support layer, immersed in a 30% KOH solution for 1 min, and then gently delaminated in DI water. The PS/MoS_2_ stack was then carefully picked up, washed, dried, and laminated onto the O_2_-plasma-treated photonic chip surface. Finally, the PS support layer was dissolved with toluene, leaving the MoS_2_ exposed for subsequent processing.

The electrical contacts were fabricated through an additional EBL step using 300 nm thick PMMA 950 A5 resist, followed by shaping the MoS_2_ by RIE with an SF_6_/O_2_ plasma. The final metallization consisted of 50 nm thick gold contacts directly onto MoS_2_, and chromium (5 nm)/gold (80 nm) contact pads deposited on HSQ using electron-beam evaporation and lift-off techniques.

### Spectroscopic Characterizations

Raman and photoluminescence (PL) spectra were collected using a Horiba LabRAM HR Evolution spectrometer equipped with a 532 nm excitation laser, operated at a low power (<0.5 mW) to prevent sample heating and oxidation. Raman spectra were acquired with an integration time of 2 s per spectrum, accumulating 50 spectra to enhance the signal-to-noise ratio, while PL measurements were performed using an integration time of 0.5 s and 2 accumulations. The excitation beam was focused onto the MoS_2_ sample using a 100× objective lens with a numerical aperture (NA) of 0.9, which also collected and collimated the scattered emission. The scattered signal was spectrally dispersed using a diffraction grating of 1800 grooves/mm (900 grooves/mm for mapping experiments), and subsequently detected by a thermoelectrically cooled charge-coupled device (CCD) detector held at −60 °C. Consistent experimental conditions (integration time, laser power, and focusing conditions) were maintained throughout all Raman and PL measurements.

### Optoelectronic Characterization

The PD optoelectronic properties were characterized using a tunable laser, a lock-in amplifier, and an optical power meter. A tunable laser, Santec TSL570, operating within the telecom C-band (1500–1600 nm), was employed as the optical source. The optical signal was internally modulated, and a lock-in amplifier, Zurich Instruments MFLI, detected the generated photovoltage. Simultaneously, the optical power was continuously monitored using a Newport 1919-R optical power meter equipped with a Newport 818-IR photodetector. The optical power coupled into the waveguide was calculated considering the fiber-to-chip coupling loss, the grating coupler insertion loss, and the Y-splitter loss. The responsivity (*R*_V_) of the photodetector was determined by dividing the measured photovoltage (*V*_ph_) by the calculated optical power within the waveguide (*P*_in_), following the relation *R*_V_ = *V*_ph_/*P*_in_. A custom MATLAB script automated the systematic measurement of the photovoltage response versus optical power, frequency response, and spectral responsivity across the C-band. All measurements were conducted at room temperature under ambient laboratory conditions.

### Noise Figure Characterization

The PDs voltage noise was measured under dark conditions using a Keysight B1500 Semiconductor Parameter Analyzer in time sampling mode. A 1.2s long voltage trace was recorded at a sampling rate of 8.33 kHz, providing a frequency resolution of ∼0.83 Hz and a Nyquist frequency of 4.165 kHz. The time-domain data was analyzed in MATLAB using a fast Fourier transform (FFT) to extract the power spectral density (PSD).

The single-sided PSD was constructed using only the positive-frequency components of the FFT. To account for the discarded negative frequencies, the squared magnitude of each FFT component (excluding DC and Nyquist) was multiplied by a factor of 2. The result was normalized by the sampling frequency and number of points, yielding the PSD in units of V^2^/Hz. The voltage noise spectral density (*v*_n_) was obtained as the square root of the PSD, and the noise equivalent power (NEP) was calculated using NEP = *v*_n_/*R*_V_, where *R*_V_ is the measured responsivity. The Johnson noise floor was estimated assuming the MoS_2_ channel resistance *R*_ch_ = 140 kΩ (see Supporting Information, Section S2), the real dissipative element to induce thermal fluctuations of $v_{n,Johnson}=\sqrt{4k_{B}TR}$ . This white noise limit appears as a flat PSD baseline at higher frequencies, while the 1/*f* flicker noise dominates at lower frequencies.

## Supplementary Material



## Notes

Additional optical, thermal, and optoelectronic simulations, MoS_2_ PL spectra and spectroscopic analysis, MoS_2_ electrical characterizations (PDF)
