# Supplementary material for: Waveguide Integrated Self-Powered MoS2 Photodetectors in the Shortwave Infrared Wavelengths
Source: ACS Photonics. 2025 Oct 20;12(11):6397–405. doi: 10.1021/acsphotonics.5c01893 (PMC12636074; doi:10.1021/acsphotonics.5c01893)
Supplement: Supplementary file 1 [file ph5c01893_si_001.pdf]

Waveguide Integrated Self-Powered MoS_2 Photodetectors in the Shortwave Infrared Wavelengths

Eitan Kaminski[1,2], Nathan Suleymanov[1], Boris Minkovich[1], Anastasios Polymerakis[3], Liana Kartvelishvili[2], Vladislav Kostianovski[2], Eilam Yalon[1], Elefterios Lidorikis[3], and Ilya Goykhman[2]*

[1]Micro Nanoelectronics Research Center, Department of Electrical and Computer Engineering, Technion, Haifa 32000, Israel

[2]Institute of Applied Physics, The Faculty of Science and The Center for Nanoscience and Nanotechnology, The Hebrew University of Jerusalem, Jerusalem 91904, Israel

[3]Department of Materials Science and Engineering, University of Ioannina, 45110, Ioannina, Greece

Supporting Information

S1. Optical Simulations

The platform used in our simulations is a silicon-on-insulator (SOI) structure, featuring a 0.22 µm-thick silicon layer and a 3 µm-thick buried oxide (BOX) layer. A planarization layer of hydrogen silsesquioxane (HSQ) with a thickness of 0.25 µm was included above the BOX. The refractive indices used in the simulations at telecom wavelengths were $n_{Si}≈3.5$ for the silicon

layer and n_{BOX}≈1.45 SiO_2/HSQ layer. We used the finite-element Lumerical commercial simulation package (MODE solver) to identify the guided optical modes supported by the waveguide structure. Specifically, a Si bus waveguide having a width and height of W=0.56 μm and $h = 0.22\ \mu m$ supports three guided modes: a transverse electric (TE), a transverse magnetic (TM) mode, and a hybrid TE/TM mode (Figure S1a). The corresponding optical effective refractive indices of the modes are presented in Figure S1b, where the values are above the refractive index of the oxide cladding to comply with the optical guiding criteria.

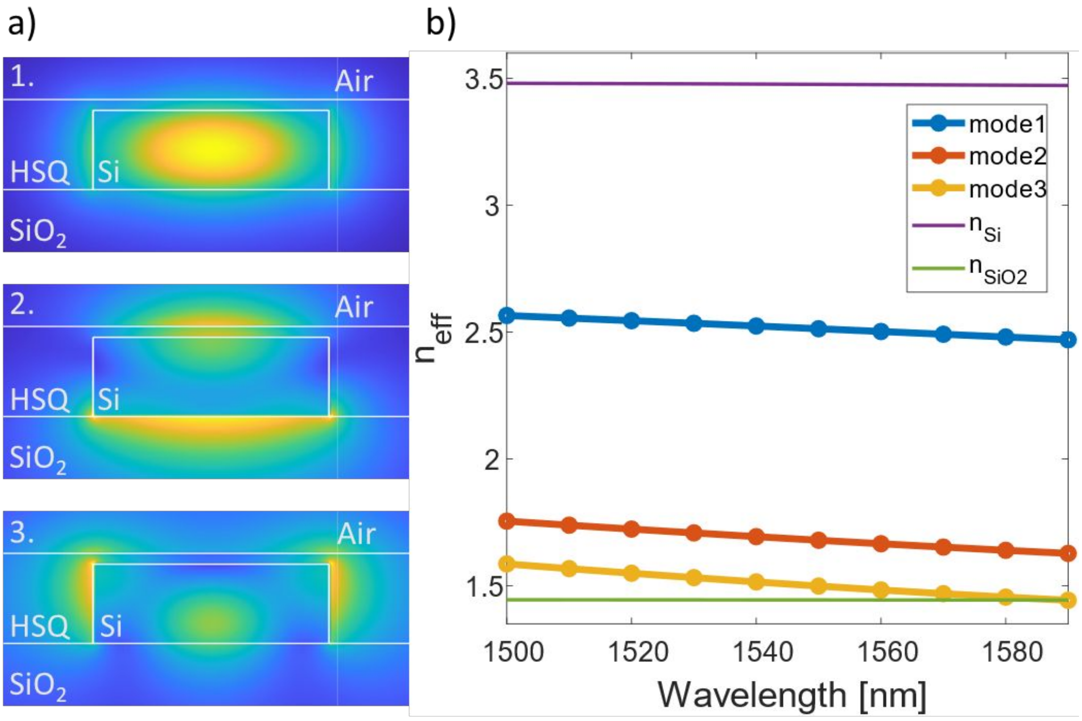

Figure S1. Mode profiles and effective refractive indices of the waveguide. (a) Electric field distributions of the three guided modes supported by the simulated SOI waveguide structure: (1) TE mode, (2) TM mode, and (3) hybrid TE/TM mode. (b) Effective refractive indices of the three modes as a function of wavelength in the C-band.

In our device, we used a Y-splitter configuration and grating couplers for light coupling. Although the grating coupler is designed to excite only the TE mode, the mode that reaches the photodetector (PD) passes through several bends along the bus waveguide, which changes its mode composition. To assess this effect, we performed 3D FDTD simulations to analyze the modal content of the optical field arriving at the PD (Figure S2a) using the mode overlap integral:

$$overlap = Re\left[\frac{\left(\int \overrightarrow{E_1} \times \overrightarrow{H_2^*} \cdot d\vec{S}\right)\left(\int \overrightarrow{E_2} \times \overrightarrow{H_1^*} \cdot d\vec{S}\right)}{\int \overrightarrow{E_1} \times \overrightarrow{H_1^*} \cdot d\vec{S}}\right] \frac{1}{Re\left[\int \overrightarrow{E_2} \times \overrightarrow{H_2^*} \cdot d\vec{S}\right]}$$

Determining the modal content at the PD input is essential for evaluating the contribution of each mode to the device's optical absorption and responsivity. At certain wavelengths, the simulated optical field distribution comprises a superposition of the first (TE) and the third (hybrid TE/TM) guided modes, as presented in Figure S2b. The total modal contribution remains normalized to unity, consistent with the completeness of the guided mode basis. The presence of higher-order modes is attributed to mode coupling when the waveguide is guided in a bent configuration and split at the Y-branch. While it equally splits the optical power between the two arms, it may not preserve modal purity, leading to variations in mode composition at the photodetector input.

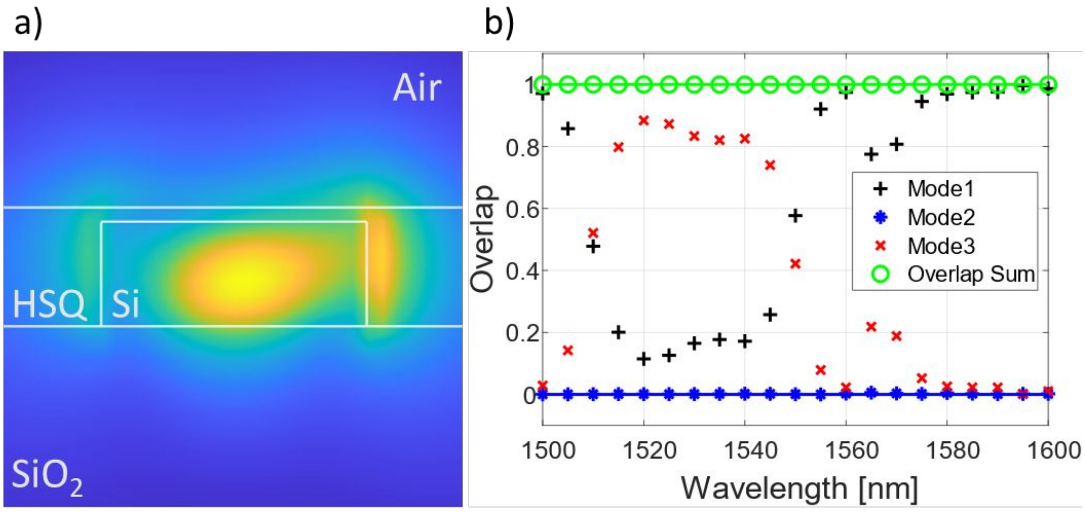

Figure S2. Mode composition at the photodetector input after propagation through the Y-splitter. (a) Simulated electric field distribution at the input to the photodetector. (b) Mode overlap analysis as a function of wavelength.

Subsequently, we used the COMSOL Multiphysics simulations in the frequency domain to calculate the optical absorption in the PD metal contact for each of the guided modes (Figure S33a). To simplify the simulations, we excluded the 1L-MoS_2 layer with a tight meshing, as it does not contribute to light absorption at telecom wavelengths. The simulation includes two gold contacts (source and drain), with the wavelength-dependent optical refractive index, e.g., $n_{Au} \approx 0.55+i11.5$ at 1550 nm wavelength. The source contact overlaps the silicon waveguide by 160nm, while the drain contact is positioned 700nm away from the waveguide. The absorption for each of the three guided modes is shown in Figure S3b. As expected, the absorption increases with wavelength for the TE mode due to its less confined nature in the waveguide. For the TM and hybrid modes, the absorption trend reverses with increasing

wavelength, likely due to the boundary conditions: TM modes tend to spread downward as the wavelength increases. Additionally, we observed that for the TE mode, the total absorption is lower compared to TM and hybrid TE/TM modes, which is consistent with the dominant in-plane electric field distribution.

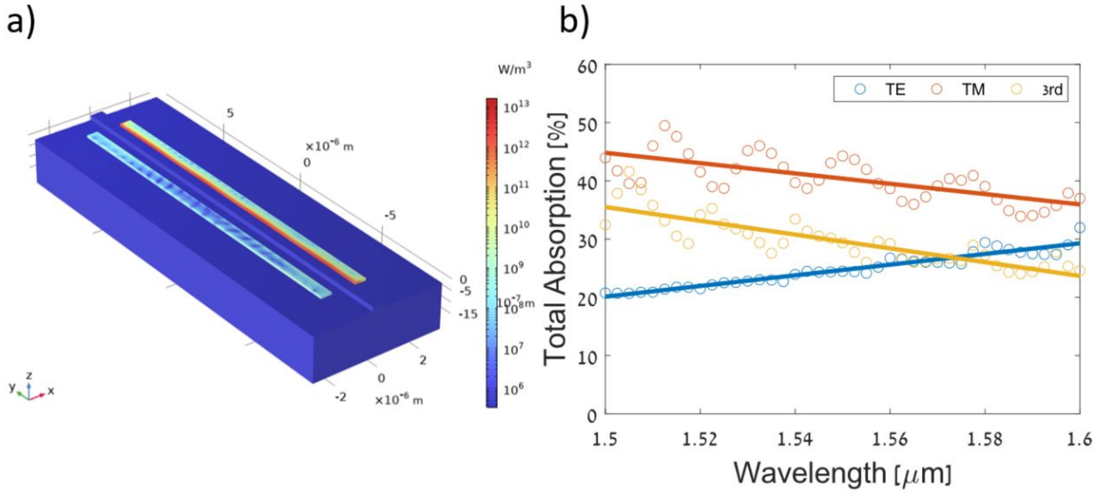

Figure S3. Optical absorption in the metal contacts for the different guided modes. (a) Simulation model showing the power dissipation density (W/m³) in the gold contacts for an incident optical mode. (b) Total absorption percentage as a function of wavelength for each of the three guided modes: TE (blue), TM (red), and the third hybrid mode (yellow).

S2. PL Analysis and Deconvolution

The room-temperature photoluminescence (PL) spectrum of monolayer MoS_2 exhibits three prominent features, corresponding to the A exciton (~664 nm, ~1.87 eV), the B exciton (~605 nm, ~2.00 eV), and the negatively charged trion A^- (~680 nm, ~1.82 eV) [9–11]. The A^- trion arises from the binding of a photoexcited electron–hole pair with an additional electron, and its formation is therefore favored by increased n-type doping [12].

To investigate the optical transitions and doping effects, we performed Lorentzian deconvolution of the PL spectra before and after device fabrication. The spectrum of as-grown MoS_2 on sapphire (Figure a) reveals three distinct peaks: ~610 nm (B exciton), ~664 nm (A exciton), and ~677 nm (A^- trion). In contrast, after the full device fabrication process (Figure b), only two features are observed: ~674 nm (A exciton) and ~689 nm (A^- trion), with the B exciton no longer detectable. The suppression of B exciton emission may arise from increased doping and the formation of trap states during transfer and fabrication, which enhance non-radiative recombination pathways [13].

The energy range and the splitting between the A exciton and A⁻ trion peaks are consistent with prior reports [9–11], validating the spectral assignment. Moreover, the relative enhancement of the trion emission post-transfer (Figure b) compared to the as-grown material (Figure a) aligns with previous studies on MoS_2 transferred onto Si/SiO_2 substrates, where the increased A^- intensity has been attributed to substrate-induced n-type doping [1].

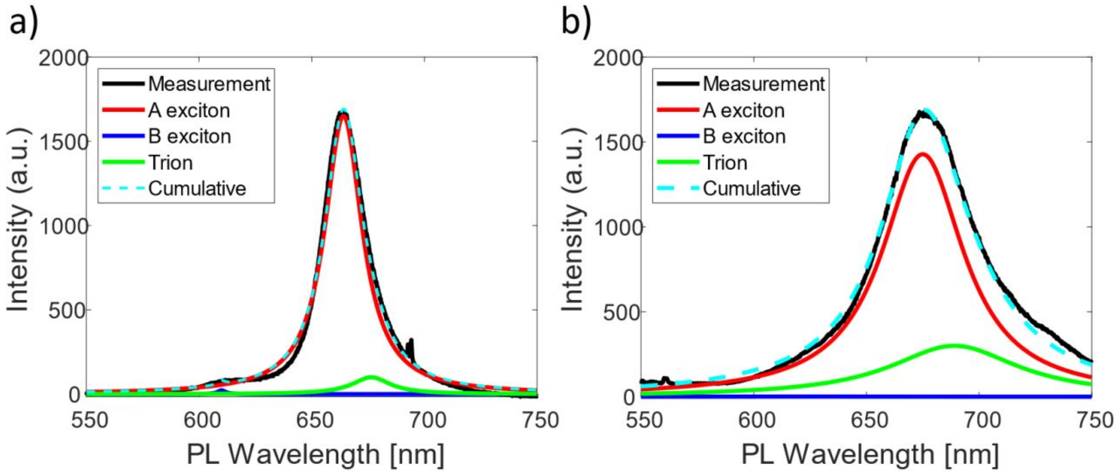

Figure S4. Lorentzian fitting of PL spectra of 1L-MoS2: (a) as-grown on sapphire and (b) after the full device fabrication process.

S3. Current-Voltage (I-V) Characterizations

Figure S shows the 2-terminal current–voltage (I–V) characterization of the photodetector measured in dark and ambient conditions. The graph highlights a non-linear I-V dependence, a Schottky junction formed at the Au/MoS2 interface. By extracting the dI/dV slope in the vicinity of zero bias (Figure S, inset), we estimate the device shunt resistance of ~500 MΩ, which includes the contributions of the 1L-MoS_2 channel and the Au/MoS_2 contact resistances at the Schottky interfaces.

To evaluate the sheet resistance of the MoS_2 channel, we used four-terminal electrical measurements, resulting in $R_{sh} \sim 6\left[\frac{M\Omega}{\square}\right]$. Given the channel length and width of $L = 25[\mu m]$ and $W = 1.5[\mu m]$ respectively, the resulting channel resistance is ~ 140 kΩ. This value is much smaller than the total 2-terminal resistance, implying that the contact resistance dominates the electrical behavior of the device at low bias.

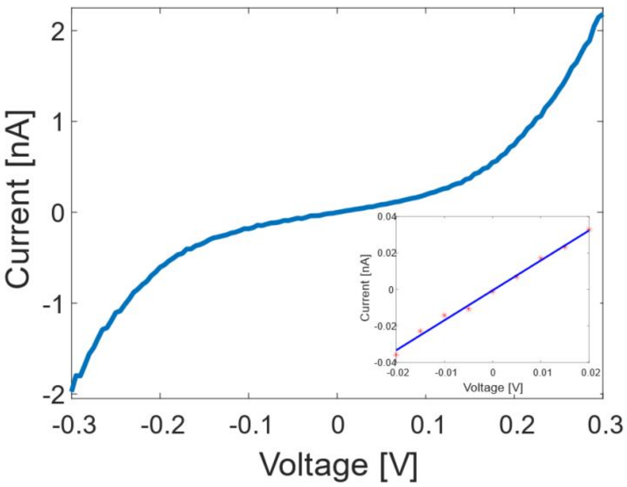

Figure S5. I–V characteristics of the photodetector measured in dark conditions and under ambient atmospheric conditions. Inset: the I-V characteristics of the device around zero bias used to extract device resistance.

S4. Heat Simulations

Following the optical simulations (Section S1), we calculated the steady-state temperature dissipation within the photodetector using COMSOL Multiphysics (Figure Sa). In this model, we incorporated a 1L-MoS_2 layer with a thickness of 0.65 nm. The thermal parameters for all materials are listed in Table S1. Interfacial thermal resistance was modelled by assigning a thermal boundary conductance (TBC) of $15 \cdot 10^6 \left[\frac{\mathrm{W}}{\mathrm{m^2K}}\right]$ for the MoS_2/SiO_2 interface [2]. A fixed room temperature boundary condition was applied at the bottom of the BOX layer. The heat source in the simulation corresponds to the optical absorption profile calculated in Section S1. Figure S shows the simulated temperature profile along the device channel for an incident optical power of 1 μW. The temperature difference between the source and drain contacts is depicted in Figure Sc.

Material	Thermal Conductivity [W/(m·K)]	Heat Capacity [J/(kg·K)]	Density [kg/m^3]
Silicon	148	711	2330
SiO_2	1.38	709	2203
Gold (Au)	178	126	19320
1L-MoS_2	34	100	5000

Table S1 Thermal parameters of the materials used in the heat transfer simulations.

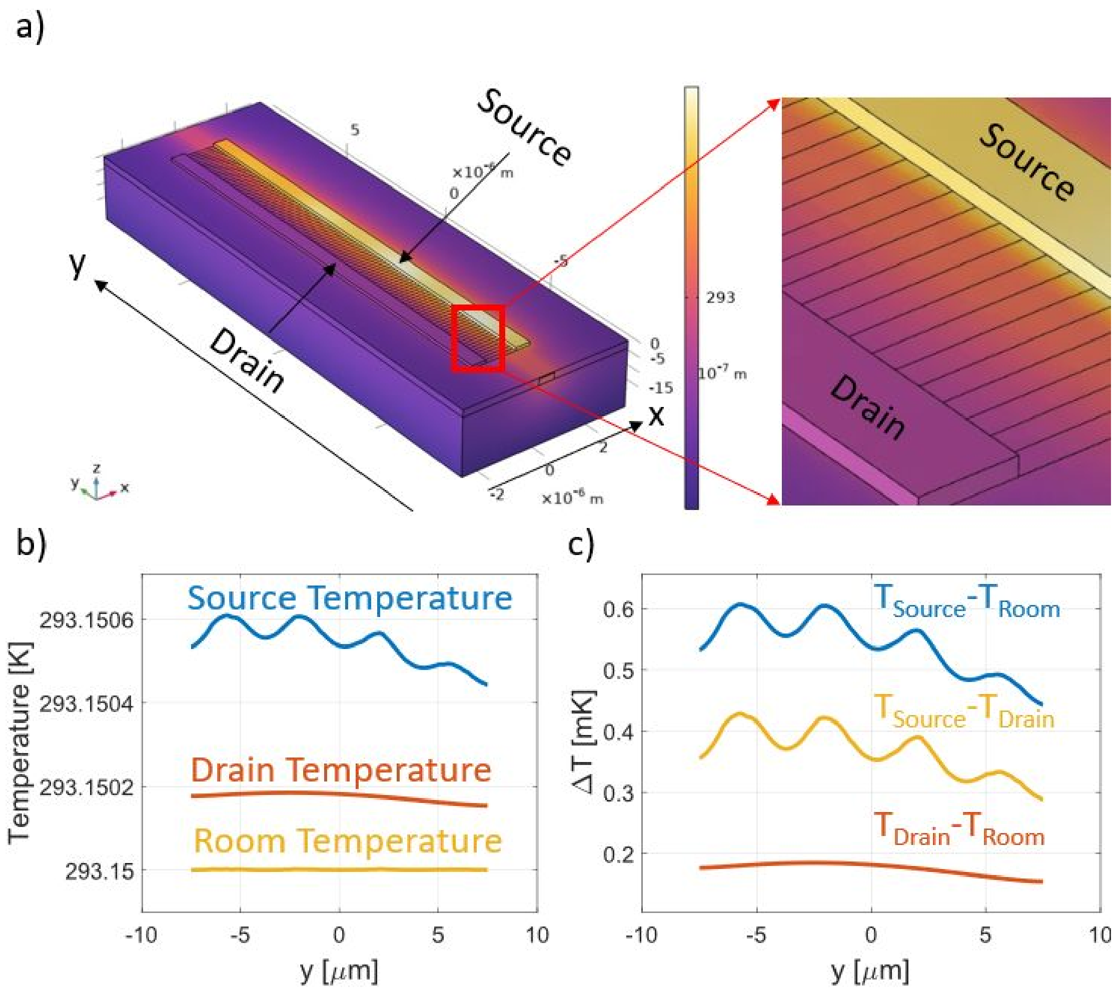

Figure S6. Steady-state thermal simulation of the photodetector. (a) Simulated temperature distribution in the device for an incident optical power of 1 μW. The right panel provides a magnified view of the heat distribution beneath the contacts. (b) The temperature profiles along the MoS_2 channel for the source, drain, and reference (room temperature) boundaries. (c) Corresponding temperature differences indicate a measurable thermal gradient across the channel that drives the photo-thermoelectric response.

S5. Photodetector Responsivity Simulations

Following the thermal simulations (Section S4), we divided the channel into multiple (hundreds) segments as illustrated in the inset of Figure Sa and computed the local temperature gradient $\frac{\partial T}{\partial x}$ between the source and drain contacts for each segment. The resulting photovoltage was calculated using the Seebeck relation:

$$V_{ph} = \int S(x)\frac{\partial T}{\partial x}dx$$

where S(x) is a spatially uniform Seebeck coefficient in the MoS_2 channel. Then, the local voltage contributions were integrated along the channel length to obtain the total photovoltage generated by the PTE effect.

In addition to the PTE effect, our device exhibits an IPE-generated photocurrent at the Au/MoS2 Schottky contact. To evaluate the IPE-induced voltage response, we employed the Fowler model, which describes the quantum efficiency (yield) of photoexcited carrier injection across a Schottky barrier. The yield Y, defined as the number of emitted photoelectrons per incident photon, is given by the Fowler equation [7]:

$$Y = A \cdot \frac{(hv - \Phi_B)^2}{(hv)^2}$$

where A is the proportionality constant dependent on materials and interface properties, hv $[eV]$ is the photon energy, and $\Phi_B[eV]$ is the Schottky barrier height of the Au/MoS_2 contact.

To compute the IPE-induced photocurrent $I_{ph}[A]$, we used the following expression:

$$I_{ph} = q \cdot Y \cdot \frac{a \cdot P}{hv}$$

where $q[e]$ is the electron charge, a is the simulated absorption fraction of incident optical power by the metal contacts, and $P[W]$ is the total incident optical power. This formulation reflects the physical interpretation that each absorbed photon contributes an excited electron, weighted by the yield. The current responsivity $R_I\left[\frac{A}{W}\right]$ can be written then:

$$R_I = \frac{I_{ph}}{P} = a \cdot \frac{q \cdot Y}{hv}$$

Finally, the voltage responsivity $R_V\left[\frac{V}{W}\right]$ is determined by multiplying the current responsivity by the load resistance of the MoS_2 channel $R_L[\Omega]$, as the photovoltage generated across the device is governed by Ohm's law:

$$R_V = R_I \cdot R_L$$

This framework provides the theoretical basis for estimating the IPE contribution to the total photoresponse. The parameter values used to match the experimental spectral response are discussed in the following section (see IPE and PTE Combination).

Finally, the device's photoresponse was modeled by combining contributions from PTE and IPE processes. To determine the overall responsivity, we weighted each mode's contribution based on its amplitude in the mode composition at the photodetector input. After applying these weights, the device's spectral response was calculated by merging the simulated outputs from both IPE and PTE effects. By fine-tuning relevant parameters within ranges supported by experimental data, we achieved a close match between the simulation results

and the measured data (Figure 4b, Main text). The key parameters used in this model are summarized in Table S2**:**

Parameter	Symbol	Value	Notes
Schottky barrier	Φ_B	$0.65[eV]$	Reported for Au/MoS_2 junctions in previous studies [8]
Proportionality constant	A	0.001%	Consistent with IPE yields in vdW heterojunctions
Load resistance	R_L	$250[M\Omega]$	Half of the total resistance was reported in the first section.
Seebeck coefficient	S	$20[mV/K]$	Within the reported range for monolayer MoS_2 [3,4]
Seebeck coefficient	S	$20[mV/K]$	Within the reported range for monolayer MoS_2 [3,4]

Table S2 Parameters used in the combined IPE and PTE model.

All chosen values fall within realistic ranges cited in the literature. The Schottky barrier height of 0.65 eV is consistent with earlier reports on Au/MoS_2 interfaces. The estimated load resistance used in the IPE calculation corresponds to half of the total shunt resistance extracted from the I–V characteristics (Section S3), reflecting the fact that the photocurrent is predominantly generated at the drain contact, while the resulting photovoltage develops primarily across the source contact. The fitting procedure highlights that both PTE and IPE processes should be considered to accurately capture the device's broadband, self-powered photoresponse.

References

[1] Yalon E, McClellan C J, Smithe K K H, Muñoz Rojo M, Xu R L, Suryavanshi S V., Gabourie A J, Neumann C M, Xiong F, Farimani A B and Pop E 2017 Energy Dissipation in Monolayer MoS2 Electronics *Nano Lett* **17** 3429–33

[2] Buscema M, Barkelid M, Zwiller V, van der Zant H S J, Steele G A and Castellanos-Gomez A 2013 Large and Tunable Photothermoelectric Effect in Single-Layer MoS2 *Nano Lett* **13** 358–63

[3] Wu J, Schmidt H, Amara K K, Xu X, Eda G and Özyilmaz B 2014 Large Thermoelectricity via Variable Range Hopping in Chemical Vapor Deposition Grown Single-Layer MoS2 *Nano Lett* **14** 2730–4

[4] Hippalgaonkar K, Wang Y, Ye Y, Qiu D Y, Zhu H, Wang Y, Moore J, Louie S G and Zhang X 2017 High thermoelectric power factor in two-dimensional crystals of MoS2 *Phys Rev B* **95** 115407

[5] Kayyalha M, Maassen J, Lundstrom M, Shi L and Chen Y P 2016 Gate-tunable and thickness-dependent electronic and thermoelectric transport in few-layer MoS2 *J Appl Phys* **120**

[6] Goykhman I, Desiatov B, Khurgin J, Shappir J and Levy U 2011 Locally Oxidized Silicon Surface-Plasmon Schottky Detector for Telecom Regime *Nano Lett* **11** 2219–24

[7] Bai H-F, Xu L-C, Di M-Y, Hao L-Y, Yang Z, Liu R P and Li X Y 2018 The intrinsic interface properties of the top and edge 1T/2H *MoS* 2 contact: A first-principles study *J Appl Phys* **123**

[8] Oz D, Suleymanov N, Minkovich B, Kostianovskii V, Gantz L, Polyushkin D, Mueller T and Goykhman I 2023 Optically Transparent and Thermally Efficient 2D MoS_2 Heaters Integrated with Silicon Microring Resonators *ACS Photonics* **10** 1783–94

[9] Lin Y, Ling X, Yu L, Huang S, Hsu A L, Lee Y-H, Kong J, Dresselhaus M S and Palacios T 2014 Dielectric Screening of Excitons and Trions in Single-Layer MoS_2 *Nano Lett* **14** 5569–76

[10] Lee H S, Kim M S, Kim H and Lee Y H 2016 Identifying multiexcitons in MoS2 monolayers at room temperature *Phys Rev B* **93** 140409

[11] Buscema M, Steele G A, van der Zant H S J and Castellanos-Gomez A 2014 The effect of the substrate on the Raman and photoluminescence emission of single-layer MoS2 *Nano Res* **7** 561–71

[12] Pandey J and Soni A 2019 Unraveling biexciton and excitonic excited states from defect bound states in monolayer MoS2 *Appl Surf Sci* **463** 52–7

[13] Liu Y, Shen T, Linghu S, Zhu R and Gu F 2022 Electrostatic control of photoluminescence from A and B excitons in monolayer molybdenum disulfide *Nanoscale Adv* **4** 2484–93
